# Impact of Sphincterotomy on Suprapapillary Inside Stent Patency in Unresectable Malignant Hilar Biliary Obstruction: A Multicenter Cohort Study

**DOI:** 10.1055/a-2962-1556

**Published:** 2026-09-28

**Authors:** So Nakaji, Kyoyoshi Saito, Emiri Kita, Wataru Yoshioka, Motoyasu Kan, Yusuke Ishida, Rintaro Mikata, Kenichi Tanaka, Toshiyasu Shiratori, Sohei Yoshimura, Hiroshi Ohyama, Toshio Tsuyuguchi, Shigenobu Yoshimura, Yoshiki Nakaya, Naoaki Tsuchiya, Hirokazu Takahashi

**Affiliations:** 1 Department of Gastroenterology 13770 Kameda Medical CenterKamogawaChibaJapan; 2 Saga Medical School Faculty of Medicine, Department of Metabolism and Endocrinology 13030 Saga UniversitySagaJapan; 3 Department of Medicine 26333 Kurume University School of MedicineKurumeJapan; 4 Department of Gastroenterology 13861 Chiba Cancer CenterChibaChiba PrefectureJapan; 5 Department of GastroenterologySaga-ken Medical Center KoseikanSagaJapan; 6 Department of Gastroenterology 12737 Chiba UniversityChibaJapan; 7 Division of Gastroenterology and Medicine 12774 Fukuoka UniversityFukuokaJapan; 8 Department of Gastroenterology 73917 Chiba Prefectural Sawara HospitalKatoriJapan; 9 Saga Medical School Faculty of Medicine, Department of Metabolism and Endocrinology, Department of Metabolism and Endocrinology 13030 Saga UniversitySagaJapan; 10 Graduate School of Medicine, Department of Gastroenterology 12737 Chiba UniversityChibaChiba PrefectureJapan; 11 Department of Gastroenterology 73917 Chiba Prefectural Sawara HospitalKatoriChibaJapan; 12 Department of Gastroenterology and Medicine 38068 Fukuoka University Faculty of MedicineFukuokaJapan

**Keywords:** pancreatobiliary (ERCP/PTCD), strictures, sphincter of Oddi manometry

## Abstract

**Background and Study Aims** The role of endoscopic sphincterotomy (EST) during suprapapillary inside stent placement for unresectable malignant hilar biliary obstruction (MHBO) remains uncertain. We evaluated its association with stent patency and adverse events.

**Patients and Methods** This multicenter retrospective cohort included 376 consecutive patients treated at 8 institutions (2014–2024). The primary outcome was time to recurrent biliary obstruction (TRBO). Multivariable Cox regression, 1:1 propensity score matching (PSM), and competing-risk analysis were performed. Patients with prior EST were excluded from index-procedure safety analyses.

**Results** Clinical success was achieved in 315 patients; 244 (77.5%) were in the EST group. TRBO did not differ between the EST and non-EST groups overall (median 170 vs 157 days; *p* = 0.85), after PSM (196 vs 207 days; *p* = 0.49), or in adjusted Cox analysis (hazard ratio 1.21, 95% CI 0.77–1.90). Competing-risk analysis likewise showed no difference (Gray’s *p* = 0.45). Among 360 patients without prior EST, PEP occurred in 7.8% versus 11.0% ( *p* = 0.388), PEB in 1.5% versus 0% ( *p* = 0.576), and perforation in 0.4% versus 0% ( *p* = 1.000) in the index EST and non-EST groups, respectively. The single perforation was type III and was classified as mild in severity.

**Conclusions** EST was not associated with longer initial stent patency. These retrospective findings do not support routine EST solely to prolong patency; its potential role in facilitating subsequent transpapillary reinterventions requires prospective evaluation.

## Introduction

Malignant hilar biliary obstruction (MHBO) is substantially more challenging to manage than distal biliary obstruction, as effective biliary drainage often requires complex, multisegmental decompression. Patients frequently experience persistent jaundice, recurrent cholangitis, and liver abscesses, all of which significantly worsen clinical outcomes. The major underlying etiologies include hilar cholangiocarcinoma, gallbladder carcinoma, hepatocellular carcinoma, and metastatic disease. For patients with unresectable MHBO, endoscopic retrograde cholangiopancreatography (ERCP)–guided biliary drainage remains the cornerstone of palliation aimed at improving symptoms and quality of life. 1 2 3

Historically, multiple plastic stents (PSs) placed across the duodenal papilla were used for drainage; however, their limited patency has largely been superseded by self-expandable metal stents (SEMS), which provide superior patency and clinical efficacy. 4 5 6 7 Multistent configurations, including side-by-side and stent-in-stent techniques, have further improved clinical outcomes. 8 9 10 Nevertheless, SEMS deployment in the hilar region is technically demanding, and reintervention remains challenging. 11

Against this background, the inside stent (suprapapillary stent) technique has gained increasing attention. By placing PS entirely within the bile duct above the papilla, this method preserves the sphincter of Oddi (SO) function and reduces duodenobiliary reflux. 12 13 14 Consequently, several studies have suggested reductions in bacterial contamination, biofilm formation, and sludge accumulation, with potential benefits including prolonged stent patency, higher reintervention success, and decreased incidence of cholangitis. 15 16 17

Despite these theoretical advantages of preserving SO function, endoscopic sphincterotomy (EST) is still routinely performed during stent placement in many centers. EST facilitates biliary access and may reduce post-ERCP pancreatitis (PEP), particularly during procedures involving intraductal ultrasound (IDUS) or tissue sampling. 18 19 20 However, EST also carries risks such as post-EST bleeding (PEB) and perforation, and the loss of SO function may paradoxically increase duodenobiliary reflux and thus shorten stent patency. 21

To date, the true clinical impact of EST in the setting of suprapapillary inside stent placement for MHBO remains unclear. Evidence is limited, and no consensus has been reached regarding whether SO preservation influences long-term patency, infectious complications, or reintervention outcomes. Likewise, it remains uncertain whether EST should be performed routinely in this context.

Given these uncertainties and the limited adjusted evidence directly comparing EST with preservation of the native papilla in both plastic and uncovered metal suprapapillary stenting, we conducted a multicenter retrospective cohort study across eight institutions. We evaluated the association of EST with time to recurrent biliary obstruction (TRBO), liver abscess, and ERCP-related adverse events using multivariable adjustment, propensity score matching, and competing-risk methods. Our aim was to provide hypothesis-generating evidence to inform individualized endoscopic strategies for unresectable MHBO.

## Patients and Methods

### Study Design

This multicenter retrospective observational study was conducted across eight institutions, with Saga University Hospital serving as the coordinating center. All consecutive patients who met the eligibility criteria and underwent endoscopic biliary drainage by inside stent placement for unresectable MHBO between April 1, 2014, and March 31, 2024, were included. The primary aim was to assess the association between EST and TRBO. No a priori sample-size or power calculation was performed because all eligible cases during the prespecified 10-year study period were included.

### Ethical Statement

This study complied with the Declaration of Helsinki and was approved by the institutional review boards of all participating institutions (registration number: 2024-10-R-04). Written informed consent for ERCP-related procedures was obtained from all patients. Consent for the use of anonymized clinical data was obtained using an opt-out approach according to national regulations.

### Patient Recruitment

Eligible patients were ≥20 years old; had unresectable MHBO caused by hilar cholangiocarcinoma, gallbladder carcinoma, hepatocellular carcinoma, or metastatic tumors; and underwent inside stent placement via ERCP. Patients initially considered unresectable but later receiving curative resection were excluded. Additional exclusion criteria included prior percutaneous transhepatic biliary drainage (PTBD), endoscopic ultrasound-guided biliary drainage (EUS-BD), or placement of a stent across the duodenal papilla. Temporary endoscopic nasobiliary drainage (ENBD) performed before inside stent placement was permitted.

### Data Collection

Clinical data were extracted from medical records at each participating institution. Retrieved variables included age, sex, primary diagnosis, Bismuth classification (types I–IV), 22 23 and postprocedural systemic chemotherapy. No standardized criteria for performing EST were shared among the participating institutions, and no institution had an explicitly defined policy requiring or prohibiting routine EST. The decision was made by the individual endoscopist based on clinical judgment and procedural strategy, including cannulation difficulty, bleeding risk, and concomitant procedures such as biliary biopsy and intraductal ultrasonography (IDUS). Patient-level stent variables included type (plastic stent [PS] or self-expandable metal stent [SEMS]), number, diameter, and extent of liver drainage (≥50% or <50% of total liver volume). Because this study included extensive hilar strictures, including Bismuth type III and IV disease, a simple unilateral-versus-bilateral classification may not accurately represent functional drainage when multiple sectoral ducts within the same hepatic lobe are involved. We therefore characterized the drainage strategy using the estimated proportion of drained liver volume (<50% or ≥50%) and the number of stents. The PSs used across the participating institutions were 7-, 8.5-, or 10-Fr Through Pass (Gadelius Medical K.K., Tokyo, Japan) or Advanix J (Boston Scientific, Marlborough, MA, USA) devices. All SEMSs were uncovered; no covered SEMSs were included. The uncovered SEMSs used across the institutions were X-Suit NIR (Olympus, Tokyo, Japan), ZeoStent V (Zeon Medical, Tokyo, Japan), Niti-S Large Cell D-type Stent (Taewoong Medical, Seoul, Korea), JOSTENT SelfX Stent (Abbott, Chicago, IL, USA), Zilver 635 (Cook Medical, Bloomington, IN, USA), BileRush Selective (Piolax Medical Devices, Yokohama, Japan), and YABUSAME (Kaneka Medical, Tokyo, Japan), with diameters of 6, 8, or 10 mm. Information on the specific stent product and stent length used in each patient, as well as the specific hepatic segments drained, was not collected in this study. Potential PEP-related variables included IDUS, cannulation time (≥10 or <10 min), biliary biopsy, pancreatic duct cannulation or contrast injection, prophylactic endoscopic pancreatic stenting (EPS), preprocedure prophylactic nonsteroidal anti-inflammatory drugs (NSAIDs), and EST.

### Statistical Analysis

All analyses were performed using R version 4.4.2 (R Foundation for Statistical Computing, Vienna, Austria). Continuous variables were compared using Student’s *t* tests, and categoric variables were compared using χ ^2^ or Fisher’s exact tests, as appropriate. Two-sided Fisher’s exact tests were used for index-procedure adverse events because event counts were sparse. Kaplan–Meier analysis was used to evaluate TRBO, with differences assessed by the log-rank test. Median TRBO and corresponding 95% confidence intervals (CIs) were derived from Kaplan–Meier estimates. A Cox proportional hazards model adjusting for age, sex, primary diagnosis, Bismuth classification, chemotherapy after stent placement, stent type, and drainage extent was used to calculate hazard ratios (HRs) with 95% CIs. To examine factors potentially associated with the decision to perform EST during the index procedure, patients with prior EST were excluded and patient-, preprocedure-, and procedure-related variables were descriptively compared between the index EST and non-EST groups using Mann–Whitney *U* tests or χ ^2^ tests with Yates’ continuity correction ( **Table S1** ). These variables were not added to the primary propensity score model because several arose during, or were selected concurrently with, the procedure and their temporal relationship to EST could not be determined retrospectively.

Propensity scores were estimated using logistic regression with the following covariates: age, sex, primary diagnosis, Bismuth classification, chemotherapy status, drainage extent, stent type, number of stents, and stent diameter. One-to-one nearest-neighbor PSM without replacement was performed using a caliper width of 0.1. Covariate balance was assessed using standardized mean differences (SMDs), with SMD < 0.1 indicating adequate balance. Post-matching TRBO was compared using the log-rank test. A Cox proportional hazards model with matched-pair clustering was also performed.

Subgroup analyses assessed whether the effect of EST on TRBO varied according to stent type (PS vs SEMS), anatomic stricture classification (Bismuth types I–II vs III–IV), or drainage extent (<50% vs ≥50% of liver volume). A two-sided *p* -value < 0.05 was considered statistically significant.

As death can preclude the occurrence of RBO, competing-risk analyses were performed with death treated as a competing event. Cumulative incidence functions (CIFs) for RBO and death were estimated and compared between the EST and non-EST groups using Gray’s test. CIF estimates at prespecified time points (90, 180, and 360 days) were summarized with 95% confidence intervals based on variance estimates obtained from the cuminc function.

Potential predictors of PEP were explored among patients without prior EST. Candidate variables included IDUS, cannulation time ≥10 min, biliary biopsy, pancreatic duct cannulation or contrast injection, prophylactic EPS, preprocedure prophylactic NSAIDs, and EST during the index treatment. Univariable associations were evaluated using χ ^2^ tests with Yates’ continuity correction. Variables with *p* < 0.10 were entered into a multivariable logistic regression model, and odds ratios (ORs) with 95% CIs were reported.

### Study Outcomes and Definitions

The primary outcome was TRBO. Recurrent biliary obstruction (RBO) was defined according to the TOKYO criteria 2014 24 as stent occlusion or migration presenting with clinical findings such as cholangitis or jaundice, supported by laboratory and/or imaging findings, and requiring biliary reintervention. The date of RBO was the date on which the first clinical manifestation attributable to stent dysfunction was identified, rather than the date of reintervention. TRBO was the interval from initial inside stent placement to RBO. Patients without RBO were censored at the last follow-up, and death before RBO was treated as a competing event. Secondary outcomes included clinical success, RBO, cholangitis, liver abscess, PEP, PEB, and perforation. Technical success was defined as placement of at least one biliary stent above the papilla without crossing the SO and was achieved in all 376 patients. This definition did not necessarily indicate successful drainage of every biliary segment initially intended, which could not be consistently determined from retrospective records. Clinical success was defined as a reduction in serum total bilirubin of at least 50% within 2 weeks or at least 75% within 4 weeks after stent placement. 25 PEP, PEB, and perforation occurring within 14 days after ERCP were diagnosed and graded according to the Cotton criteria. 26 Cholangitis and liver abscess were assessed throughout follow-up; cholangitis was diagnosed according to the TOKYO criteria 2014, 24 and liver abscess was diagnosed from clinical, laboratory, and imaging findings. Patients with prior EST were excluded from analyses of index-procedure PEP, PEB, perforation, and PEP risk factors because EST was not performed during the index treatment in these patients.

## Results

### Patient Characteristics

A total of 376 patients underwent technically successful inside stent placement for unresectable MHBO (technical success, 100%). Clinical success was achieved in 315 patients, who were included in the primary TRBO analysis ( Fig. 1 ). Their baseline characteristics are summarized in Table 1 . The mean age was 72.9 years, and 60.0% were male. The most frequent diagnosis was hilar cholangiocarcinoma (46.0%), followed by gallbladder carcinoma (24.4%), hepatocellular carcinoma (8.6%), and other malignancies (21.0%). Bismuth type III–IV strictures accounted for 71.4% of cases. EST had been performed in 244 patients (77.5%). PSs were used in 54.3% and uncovered SEMSs in 45.7%. The mean follow-up duration was 271.5 days.

**Fig. 1:**
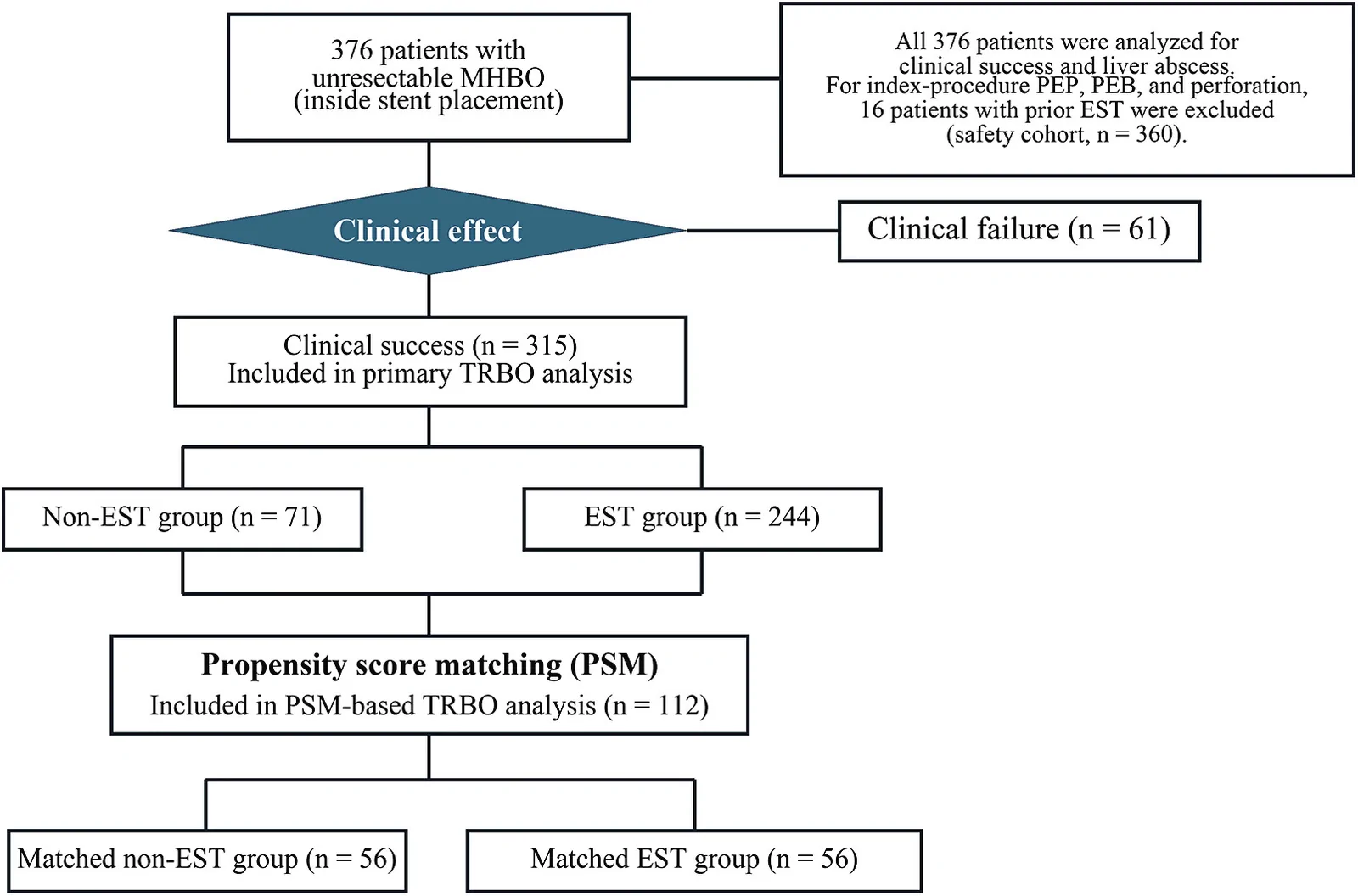
Patient flow diagram. A total of 376 patients who underwent suprapapillary inside stent placement for unresectable malignant hilar biliary obstruction were assessed. Clinical success was achieved in 315 patients, who were included in the primary analysis of time to recurrent biliary obstruction: 244 in the EST group and 71 in the non-EST group. After 1:1 propensity score matching, 56 matched pairs were included in the adjusted analysis. All 376 patients were assessed for clinical success and liver abscess. For the analyses of index-procedure post-ERCP pancreatitis, post-EST bleeding, and perforation, 16 patients with prior EST were excluded, leaving a safety cohort of 360 patients. EST, endoscopic sphincterotomy; ERCP, endoscopic retrograde cholangiopancreatography.

**Table 1:** Baseline characteristics of the study population and comparison between the EST and non-EST groups before and after propensity score matching.

		Overall	Unmatched group				Matched group			
Factor	Group	Overall	Non-EST	EST	*p* -Value	SMD	Non-EST	EST	*p* -Value	SMD
n		315	71	244			56	56		
Age (mean (SD))		72.88 (11.68)	73.07 (12.82)	72.82 (11.35)	0.874	0.021	72.41 (13.62)	71.18 (11.53)	0.606	0.098
Sex (%)	F	126 (40.0)	33 (46.5)	93 (38.1)	0.259	0.17	25 (44.6)	28 (50.0)	0.705	0.107
	M	189 (60.0)	38 (53.5)	151 (61.9)			31 (55.4)	28 (50.0)		
Primary diagnosis (%)	Hilar cholangiocarcinoma	145 (46.0)	25 (35.2)	120 (49.2)	0.047	0.363	23 (41.1)	20 (35.7)	0.899	0.145
	Gallbladder carcinoma	77 (24.4)	20 (28.2)	57 (23.4)			15 (26.8)	17 (30.4)		
	Hepatocellular carcinoma	27 (8.6)	11 (15.5)	16 (6.6)			5 (8.9)	4 (7.1)		
	Others	66 (21.0)	15 (21.1)	51 (20.9)			13 (23.2)	15 (26.8)		
Bismuth classification (%)	I or II	90 (28.6)	19 (26.8)	71 (29.1)	0.815	0.052	15 (26.8)	20 (35.7)	0.415	0.194
	III or IV	225 (71.4)	52 (73.2)	173 (70.9)			41 (73.2)	36 (64.3)		
Chemotherapy after stent placement (%)	−	145 (46.0)	39 (54.9)	106 (43.4)	0.116	0.231	28 (50.0)	29 (51.8)	1	0.036
	+	170 (54.0)	32 (45.1)	138 (56.6)			28 (50.0)	27 (48.2)		
Drainage extent (%)	≥50%	223 (70.8)	57 (80.3)	166 (68.0)	0.064	0.283	44 (78.6)	43 (76.8)	1	0.043
	<50%	92 (29.2)	14 (19.7)	78 (32.0)			12 (21.4)	13 (23.2)		
Stent type (%)	PS	171 (54.3)	18 (25.4)	153 (62.7)	<0.001	0.812	15 (26.8)	15 (26.8)	1	<0.001
	SEMS	144 (45.7)	53 (74.6)	91 (37.3)			41 (73.2)	41 (73.2)		
Number of stents (%)	1	168 (53.3)	27 (38.0)	141 (57.8)	<0.001	0.531	26 (46.4)	30 (53.6)	0.597	0.193
	2	136 (43.2)	36 (50.7)	100 (41.0)			26 (46.4)	24 (42.9)		
	3	11 (3.5)	8 (11.3)	3 (1.2)			4 (7.1)	2 (3.6)		
Stent diameter (%)	7 Fr	152 (48.3)	16 (22.5)	136 (55.7)	<0.001	1.064	15 (26.8)	15 (26.8)	–	0.131
	8.5 Fr	17 (5.4)	0 (0.0)	17 (7.0)			0 (0.0)	0 (0.0)		
	6 Fr and 7 Fr	2 (0.6)	2 (2.8)	0 (0.0)			0 (0.0)	0 (0.0)		
	6 mm	7 (2.2)	0 (0.0)	7 (2.9)			0 (0.0)	0 (0.0)		
	8 mm	90 (28.6)	30 (42.3)	60 (24.6)			25 (44.6)	28 (50.0)		
	10 mm	45 (14.3)	21 (29.6)	24 (9.8)			16 (28.6)	13 (23.2)		
	6 mm and 8 mm	1 (0.3)	1 (1.4)	0 (0.0)			0 (0.0)	0 (0.0)		
	8 mm and 10 mm	1 (0.3)	1 (1.4)	0 (0.0)			0 (0.0)	0 (0.0)		

EST, endoscopic sphincterotomy; PS, plastic stent; SEMS, self-expandable metal stent; SMD, standardized mean difference.

### Comparison between EST and Non-EST Groups before and after Propensity Score Matching

Before PSM, several characteristics differed between the EST and non-EST groups, including primary diagnosis, stent type, number of stents, and stent diameter ( Table 1 ). After 1:1 PSM using age, sex, primary diagnosis, Bismuth classification, chemotherapy status, drainage extent, stent type, number of stents, and stent diameter as covariates, 56 matched pairs were generated and the measured baseline characteristics were well balanced. In the descriptive analysis excluding 16 patients with prior EST, the non-EST and index EST groups included 91 and 269 patients, respectively ( **Table S1** ). Precut, biliary biopsy, and pre-procedure prophylactic NSAID use were more frequent in the index EST group, whereas pancreatic duct cannulation or contrast injection and prophylactic EPS were more frequent in the non-EST group. IDUS and cannulation time ≥10 min did not differ significantly.

### Overall Clinical Outcomes and ERCP-related Adverse Events

Clinical outcomes are shown in Table 2 . Among all 376 patients, clinical success was achieved in 85.6% of the EST group and 78.0% of the non-EST group ( *p* = 0.089). Among the 315 patients with clinical success, RBO occurred in 114 of 244 patients (46.7%) in the EST group and 41 of 71 (57.7%) in the non-EST group ( *p* = 0.108). Liver abscess was assessed among all 376 patients and occurred in 4.2% of the EST group and 4.4% of the non-EST group ( *p* = 1.000); its location did not differ significantly ( *p* = 0.264). After excluding 16 patients with prior EST, index-procedure safety analyses included 360 patients (269 index EST and 91 non-EST). PEP occurred in 21 patients (7.8%) in the index EST group and 10 (11.0%) in the non-EST group ( *p* = 0.388); severity distributions were similar ( *p* = 0.634). PEB occurred in four patients (1.5%) after index EST and none without EST ( *p* = 0.576). One type III perforation, classified as mild in severity, occurred after index EST (0.4%), whereas none occurred without EST ( *p* = 1.000).

**Table 2:** Clinical outcomes and ERCP-related adverse events.

Factor	Non-EST	EST	*p* -Value
*n* (all cases)	91	285	
Clinical success (%)	71 (78.0)	244 (85.6)	0.089
RBO among patients with clinical success (%)	41 (57.7)	114 (46.7)	0.108
Liver abscess (%)	4 (4.4)	12 (4.2)	1.000
Abscess location (%)			0.264
Drained area	2 (50.0)	6 (50.0)	
Undrained area	2 (50.0)	2 (16.7)	
Mixed	0 (0.0)	4 (33.3)	
n (without prior EST cases)	91	269	
Post-ERCP pancreatitis (PEP), *n* (%)	10 (11.0)	21 (7.8)	0.388
Mild, *n* (%)	9 (90.0)	16 (76.2)	0.634
Moderate, *n* (%)	1 (10.0)	5 (23.8)	
Post-EST bleeding (PEB), *n* (%)	0 (0.0)	4 (1.5)	0.576
Mild, *n* (%)	0	2 (50.0)	
Moderate, *n* (%)	0	1 (25.0)	
Severe, *n* (%)	0	1 (25.0)	
Perforation, *n* (%)	0 (0.0)	1 (0.4)	1.000
Mild, *n* (%)	0	1 (100)	

Data are presented as *n* (%). RBO was assessed among the 315 patients with clinical success (non-EST, *n* = 71; EST, *n* = 244). Liver abscess was assessed among all 376 patients. Patients with prior EST ( *n* = 16) were excluded from the analyses of PEP, PEB, and perforation because these adverse events were evaluated according to whether EST was performed at the index treatment.

### Time to Recurrent Biliary Obstruction (TRBO)

Kaplan–Meier analysis of clinically successful cases demonstrated no significant difference in TRBO between the EST and non-EST groups (median: 170 vs. 157 days; log-rank *p* = 0.85; Fig. 2 ). After PSM, TRBO remained comparable between groups (median: 196 vs. 207 days; log-rank *p* = 0.486; Fig. 3 ). A Cox proportional hazards model incorporating matched-pair clustering similarly showed no association between EST and TRBO (HR 1.21, 95% CI 0.77–1.90; *p* = 0.41).

**Fig. 2:**
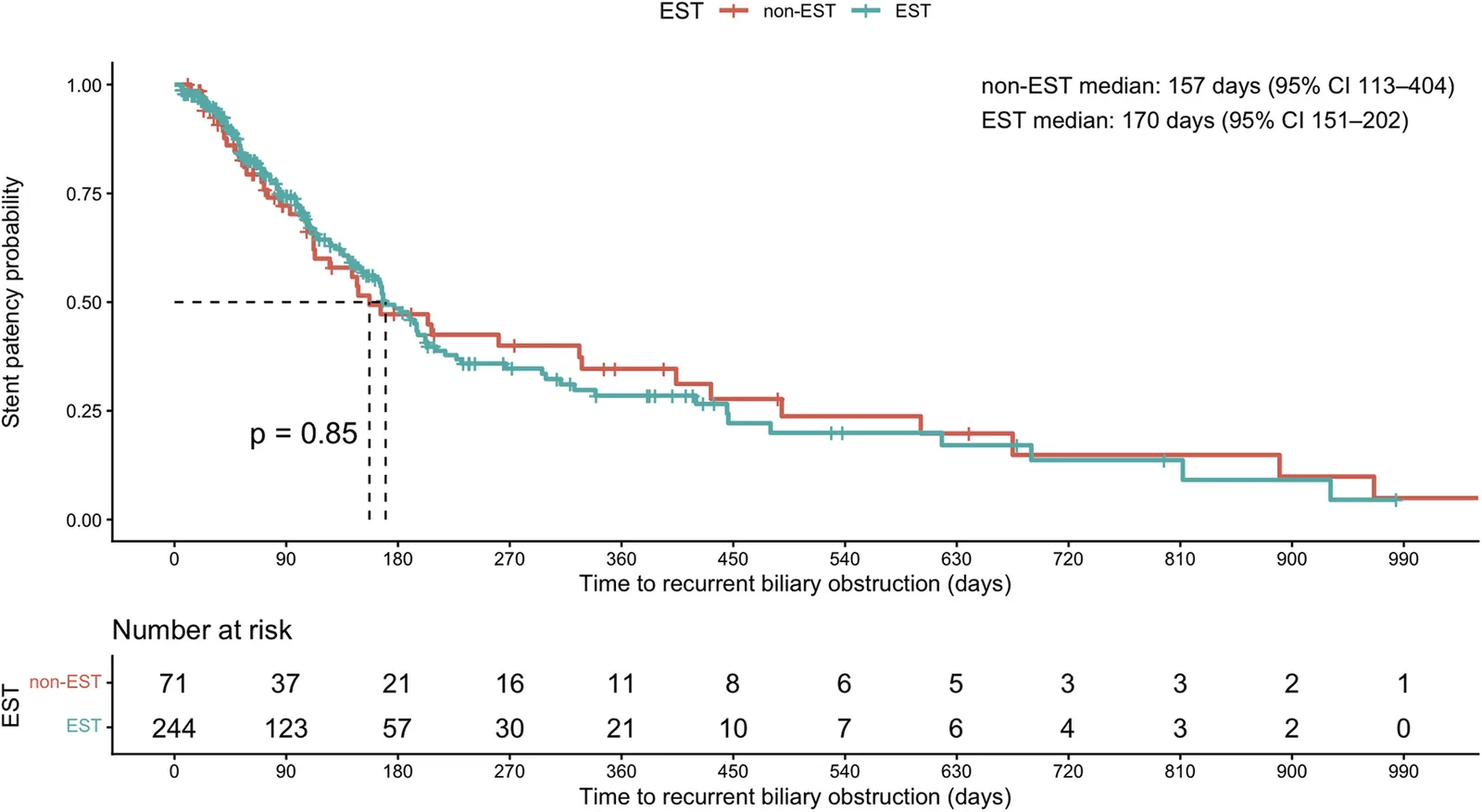
Kaplan–Meier curves for time to recurrent biliary obstruction in clinically successful cases according to endoscopic sphincterotomy status. The median time to recurrent biliary obstruction was 170 days (95% CI 151–202) in the EST group and 157 days (95% CI 113–404) in the non-EST group (log-rank *p* = 0.85). Numbers at risk are shown below the graph. EST, endoscopic sphincterotomy; CI, confidence interval.

**Fig. 3:**
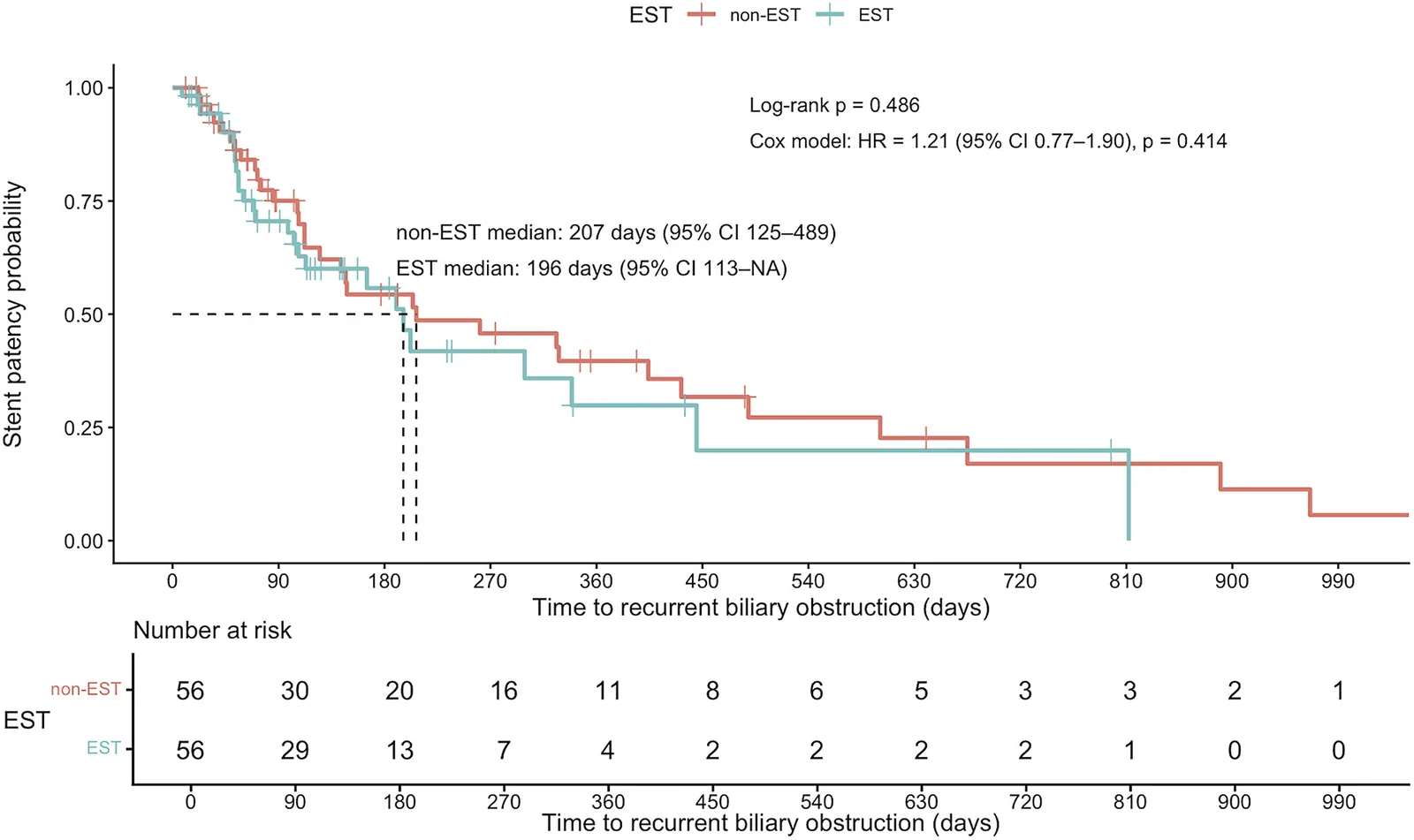
Kaplan–Meier curves for time to recurrent biliary obstruction after 1:1 propensity score matching according to endoscopic sphincterotomy status. Fifty-six matched pairs were analyzed. The median time to recurrent biliary obstruction was 196 days (95% CI 113–not estimable) in the EST group and 207 days (95% CI 125–489) in the non-EST group (log-rank *p* = 0.486). A Cox proportional hazards model accounting for matched-pair clustering showed no significant association between EST and time to recurrent biliary obstruction (HR for EST vs. non-EST 1.21, 95% CI 0.77–1.90; *p* = 0.414). Numbers at risk are shown below the graph. EST, endoscopic sphincterotomy; HR, hazard ratio; CI, confidence interval.

Competing-risk analysis treating death as a competing event similarly demonstrated no difference in the cumulative incidence of RBO between groups (Gray’s test *p* = 0.45). The cumulative incidence of RBO at 90, 180, and 360 days was 0.226 (95% CI 0.171–0.281), 0.394 (0.329–0.459), and 0.490 (0.423–0.556) in the EST group and 0.248 (0.145–0.351), 0.415 (0.296–0.535), and 0.491 (0.370–0.613) in the non-EST group, respectively. The cumulative incidence of death did not differ significantly between groups ( *p* = 0.238), with 360-day estimates of 0.406 (0.340–0.471) in the EST group and 0.341 (0.226–0.457) in the non-EST group ( **Fig. S1** and **Table S2** ).

### Subgroup Analyses

Across all subgroup analyses, no beneficial effect of EST on TRBO was observed. Among patients treated with SEMS, TRBO did not differ between the EST and non-EST groups (median: 202 vs. 261 days; *p* = 0.80; **Fig. S2** ). Similarly, no differences were observed among those receiving PS (median: 161 vs. 147 days; *p* = 0.40; **Fig. S3** ). When stratified by stricture anatomy, TRBO was comparable in patients with Bismuth types I–II (median: 154 vs. 85 days; *p* = 0.40; **Fig. S4** ) and in those with types III–IV (median: 182 vs. 157 days; *p* = 0.60; **Fig. S5** ). Drainage adequacy did not modify the effect of EST: TRBO did not differ in cases with <50% drainage (median: 202 vs. 147 days; *p* = 0.80; **Fig. S6** ) or ≥50% drainage (median: 161 vs. 166 days; *p* = 0.60; **Fig. S7** ). In contrast, an exploratory analysis independent of EST showed that SEMS exhibited significantly longer stent patency than PS (median: 231 vs. 151 days; *p* = 0.03; **Fig. S8** ).

### Risk Factors for PEP

Among 360 patients without prior EST, univariable analysis identified cannulation time ≥10 min and pancreatic duct cannulation or contrast injection as factors associated with PEP, while biliary biopsy showed a nonsignificant trend. In the complete-case multivariable model ( *n* = 359), none reached statistical significance: cannulation time ≥10 min, OR 2.00 (95% CI 0.86–4.64; *p* = 0.106); biliary biopsy, OR 1.90 (95% CI 0.89–4.03; *p* = 0.095); and pancreatic duct cannulation or contrast injection, OR 1.96 (95% CI 0.87–4.40; *p* = 0.104) ( **Table S3** ).

## Discussion

This multicenter retrospective study provides hypothesis-generating evidence on EST during suprapapillary inside stent placement for unresectable MHBO. Among the 315 patients who achieved clinical success, median TRBO was 170 days in the EST group and 157 days in the non-EST group (log-rank *p* = 0.85); after 1:1 PSM, the corresponding medians were 196 and 207 days ( *p* = 0.486), and the matched Cox model showed no association between EST and TRBO (HR 1.21, 95% CI 0.77–1.90; *p* = 0.41). RBO occurred in 114 of 244 patients (46.7%) in the EST group and 41 of 71 patients (57.7%) in the non-EST group ( *p* = 0.108). EST was likewise not associated with TRBO in the multivariable, competing-risk, or subgroup analyses. The consistency across complementary methods is a strength, but it should not be interpreted as proof of equivalence or causality because the study was observational, not powered for a prespecified effect, and remained susceptible to residual confounding.

Given the substantial mortality in unresectable MHBO, we evaluated RBO using competing-risk methods that account for death as a competing event. 27 At 360 days, the cumulative incidence of RBO was 0.490 (95% CI 0.423–0.556) in the EST group and 0.491 (95% CI 0.370–0.613) in the non-EST group, with no significant difference through 360 days (Gray’s *p* = 0.45). This finding supports the consistency of the observed null association across Kaplan–Meier, Cox, and competing-risk frameworks.

Inside stents were designed to reduce duodenobiliary reflux, bacterial contamination, biofilm formation, and sludge accumulation. Uchida et al. 28 reported longer patency with suprapapillary than transpapillary Tannenbaum stents (median 255 vs. 82 days; *p* < 0.01). In MHBO, Kurita et al. 16 showed longer patency for suprapapillary inside PSs placed without EST than for conventional PSs (median 123 vs. 51 days; *p* = 0.031). In a prospective multicenter registry of 81 patients with malignant biliary strictures, Kogure et al. 29 reported a median TRBO of 4.7 months and no significant difference in patency according to EST or balloon dilation. Kanno et al. 30 likewise found no significant overall difference according to EST in an RCT subanalysis ( *p* = 0.62); the corresponding median values were 149 vs. 83 days for inside PSs and 110 vs. 447 days for inside metal stents, with no significant subgroup difference ( *p* = 0.40). Our study adds a larger multicenter cohort restricted to unresectable MHBO, includes both PSs and uncovered SEMSs, and evaluates the association using multivariable adjustment, PSM, subgroup analysis, and death as a competing risk. Together, these data suggest that any effect of papillary physiology on initial stent patency may be limited when the distal stent end remains above the papilla; however, the present retrospective analysis cannot establish equivalence.

In contrast, distal malignant biliary obstruction represents a different anatomic and physiologic setting. Takada et al. 31 reported longer patency with intraductal than transpapillary SEMSs (median 307 vs. 161 days; *p* = 0.01), and the longest patency when EST was omitted within the intraductal group (363 vs. 227 days; *p* = 0.04). As the papilla lie immediately downstream from a distal obstruction, loss of sphincter function may have a greater effect on reflux and stent dysfunction than in MHBO.

Among the 360 patients without prior EST, who were included in the index-procedure safety analysis, PEP occurred in 21 of 269 patients (7.8%) in the index EST group and 10 of 91 patients (11.0%) in the non-EST group ( *p* = 0.388). PEB occurred only after EST (4/269, 1.5% vs. 0/91; *p* = 0.576), and one mild type III perforation occurred after EST (1/269, 0.4% vs. 0/91; *p* = 1.000), although sparse events preclude precise risk estimates. Kato et al. 32 reported a significantly lower incidence of PEP with EST during transpapillary drainage (3.9% vs. 20.6%; *p* < 0.001), in which devices traverse the papilla and can affect pancreatic outflow. That setting differs from suprapapillary inside stenting. Accordingly, our data do not demonstrate a PEP-preventive benefit of EST in this setting and identify potential harms that should be balanced against technical needs.

The relatively high PEP incidence may reflect the complexity of MHBO procedures, which often require prolonged cannulation, pancreatic duct instrumentation, tissue sampling, IDUS, and repeated device passage. Differences in patient selection, disease extent, procedural complexity, preventive practice, institutional policy, and endoscopist strategy may also have contributed. In the analysis excluding patients with prior EST, pancreatic duct cannulation or contrast injection was more frequent in the non-EST group, whereas prophylactic NSAID administration was more frequent in the index EST group. These imbalances complicate a simple interpretation of PEP incidence according to EST status. In the complete-case multivariable model ( *n* = 359), cannulation time ≥10 min (OR 2.00, 95% CI 0.86–4.64; *p* = 0.106), biliary biopsy (OR 1.90, 95% CI 0.89–4.03; *p* = 0.095), and pancreatic duct cannulation or contrast injection (OR 1.96, 95% CI 0.87–4.40; *p* = 0.104) showed elevated point estimates, but none reached statistical significance. This exploratory analysis did not establish that EST prevents PEP. PEP was not the primary outcome, and the study was not designed or powered to detect a difference in PEP incidence. As only 31 PEP events occurred among the 360 patients included in the safety analysis, statistical precision was limited, and a clinically relevant association may have remained undetected because of insufficient power. Therefore, the absence of statistical significance should not be interpreted as evidence of no difference or equivalence between the groups. The absence of an association between EST and initial TRBO does not imply that EST has no clinical value in every setting. Although our findings did not support routine EST solely to prolong initial stent patency, EST may have a technical role in selected patients. In particular, when RBO or cholangitis occurs, EST may facilitate subsequent transpapillary biliary access and the removal or exchange of an inside stent. EST may also be required for a specific technical indication, such as precut sphincterotomy during difficult biliary cannulation. These potential benefits must be balanced against procedure-related adverse events. However, our study did not evaluate the success rate, procedure time, or technical difficulty of subsequent reinterventions. Therefore, the present data cannot establish that EST improves the outcomes of subsequent reintervention. Prospective studies are needed to determine which patients may benefit from selective EST.

This study has several strengths, including consecutive enrollment across eight institutions, a clinically relevant MHBO cohort, standardized definitions, and consistent findings across multivariable, propensity-matched, subgroup, and competing-risk analyses. The use of a variety of PSs and uncovered SEMSs across the participating institutions may also improve the applicability of the findings to different real-world inside-stenting strategies rather than limiting them to a single stent product. Important limitations remain. First, no standardized EST criteria were used, the specific rationale was not consistently documented, and imbalances in patient-, preprocedure-, and procedure-related factors indicate possible confounding by indication. Several procedural variables were not added to the primary propensity model because their temporal relationship to EST was unclear; unmeasured and residual confounding therefore remain. Second, no a priori sample-size calculation was performed, enrolment was limited and uneven across institutions, and the matched sample and infrequent adverse-event counts limited statistical precision and power. In particular, the PEP analysis may have been underpowered to detect a clinically relevant association. Third, stent selection and drainage strategy varied. Although stent type, diameter, number, and estimated drainage extent were collected, information on the specific stent product and length used in each patient and the specific hepatic segments drained was not collected. Consequently, these factors could not be evaluated or fully adjusted for and may have resulted in the residual confounding. Thus, device diversity may enhance real-world applicability while also introducing uncontrolled heterogeneity. Fourth, reintervention success, difficulty, and procedure time were not evaluated. Finally, devices, chemotherapy, preventive practice, and endoscopist experience changed over the 10-year period. These limitations make the findings preliminary rather than definitive.

In this multicenter retrospective cohort, EST was not associated with initial stent patency during suprapapillary inside stent placement for unresectable MHBO. These findings do not support routine EST solely to prolong initial patency. EST may nevertheless facilitate subsequent transpapillary reinterventions in selected patients, an outcome not evaluated in this study. Prospective studies are warranted to clarify the optimal indications for EST.

## Declaration of GenAI Use

The authors used ChatGPT and Codex (OpenAI) for English-language editing, translation, and assistance in organizing responses to the reviewers. The authors critically reviewed and revised all AI-assisted content and take full responsibility for the accuracy, integrity, and final content of the manuscript.

## Supplementary Material

Ergänzendes Material Supplementary Material
