# Supplementary material for: Impact of Sphincterotomy on Suprapapillary Inside Stent Patency in Unresectable Malignant Hilar Biliary Obstruction: A Multicenter Cohort Study
Source: Endosc Int Open. 2026 Sep 28;14:a29621556. doi: 10.1055/a-2962-1556 (PMC13643726; doi:10.1055/a-2962-1556)

Table S1. Exploratory descriptive comparison of patient-, pre-procedure-, and procedure-related characteristics between the non-EST and index EST groups among patients without prior EST.

**Factor**	**Non-EST (n=91)**	**EST at index treatment (n=269)**	**p-value**
Liver cirrhosis, Child–Pugh class B or C	9 (9.9)	13 (4.8)	0.137
Hemodialysis	1 (1.1)	0 (0.0)	0.569
Antithrombotic therapy	10 (11.0)	23 (8.6)	0.626
Precut sphincterotomy	0 (0.0)	18 (6.7)	0.024
Platelet count, $\times 10^4/\mu L$	20.1 (13.3–27.4)	23.4 (18.5–29.3)	0.027
PT-INR	1.06 (0.96–1.16)	1.01 (0.93–1.10)	0.019
Pancreatic duct cannulation or contrast injection	55 (60.4)	91 (33.8)	<0.001
Cannulation time ≥10 min	36 (40.0)	129 (48.0)	0.235
Biliary biopsy	24 (26.4)	104 (38.7)	0.047
Intraductal ultrasonography	7 (7.7)	23 (8.6)	0.971
Prophylactic pancreatic duct stenting	25 (27.8)	13 (4.8)	<0.001
Prophylactic NSAID administration	6 (6.6)	112 (41.6)	<0.001

PT-INR, prothrombin time–international normalized ratio; EST, endoscopic sphincterotomy.

Table S2. Cumulative incidence function (CIF) estimates for recurrent biliary obstruction (RBO) and death at prespecified time points (90, 180, and 360 days) according to endoscopic sphincterotomy (EST) status.

Outcome	Group	90 days (95%CI)	180 days (95%CI)	360 days (95%CI)
RBO	Non-EST	0.248 (0.145-0.351)	0.415 (0.296-0.535)	0.491 (0.370-0.613)
RBO	EST	0.226 (0.171-0.281)	0.394 (0.329-0.459)	0.490 (0.423-0.556)
Death	Non-EST	0.189 (0.096-0.283)	0.265 (0.159-0.372)	0.341 (0.226-0.457)
Death	EST	0.223 (0.168-0.277)	0.334 (0.272-0.397)	0.406 (0.340-0.471)

CI, confidence interval; CIF, cumulative incidence function; EST, endoscopic sphincterotomy; RBO, recurrent biliary obstruction.

Table S3. Univariable and multivariable analyses of risk factors for post-ERCP pancreatitis in patients without prior EST undergoing suprapapillary inside stent placement.

Factor	Level	Overall	Post-ERCP pancreatitis		Univariate analysis	Multivariate analysis	
			−	+	p-value	OR (95% CI)	p-value
n		360	329	31			
Intraductal ultrasound (IDUS) (%)	−	330 (91.7)	303 (92.1)	27 (87.1)	0.533		
	+	30 (8.3)	26 (7.9)	4 (12.9)			
Cannulation time ≥10 min (%)	−	194 (54.0)	184 (56.1)	10 (32.3)	0.018	2.00 (0.86–4.64)	0.106
	+	165 (46.0)	144 (43.9)	21 (67.7)			
Biliary biopsy (%)	−	232 (64.4)	217 (66.0)	15 (48.4)	0.079	1.90 (0.89–4.03)	0.095
	+	128 (35.6)	112 (34.0)	16 (51.6)			
Pancreatic duct cannulation/contrast (%)	−	214 (59.4)	202 (61.4)	12 (38.7)	0.023	1.96 (0.87–4.40)	0.104
	+	146 (40.6)	127 (38.6)	19 (61.3)			
Prophylactic pancreatic duct stenting (%)	−	321 (89.4)	295 (89.9)	26 (83.9)	0.457		
	+	38 (10.6)	33 (10.1)	5 (16.1)			
Prophylactic NSAIDs (%)	−	242 (67.2)	220 (66.9)	22 (71.0)	0.791		
	+	118 (32.8)	109 (33.1)	9 (29.0)			
EST at index treatment (%)	−	91 (25.3)	81 (24.6)	10 (32.3)	0.472		
	+	269 (74.7)	248 (75.4)	21 (67.7)			

IDUS, Intraductal ultrasound; EST, endoscopic sphincterotomy; NSAIDs, nonsteroidal anti-inflammatory drugs.

Percentages were calculated using available data. One value each was missing for cannulation time and prophylactic pancreatic duct stenting.

Figure S1. Cumulative incidence function curves for recurrent biliary obstruction and death according to endoscopic sphincterotomy status. / CIFs for RBO and death are displayed in a single panel up to 720 days.

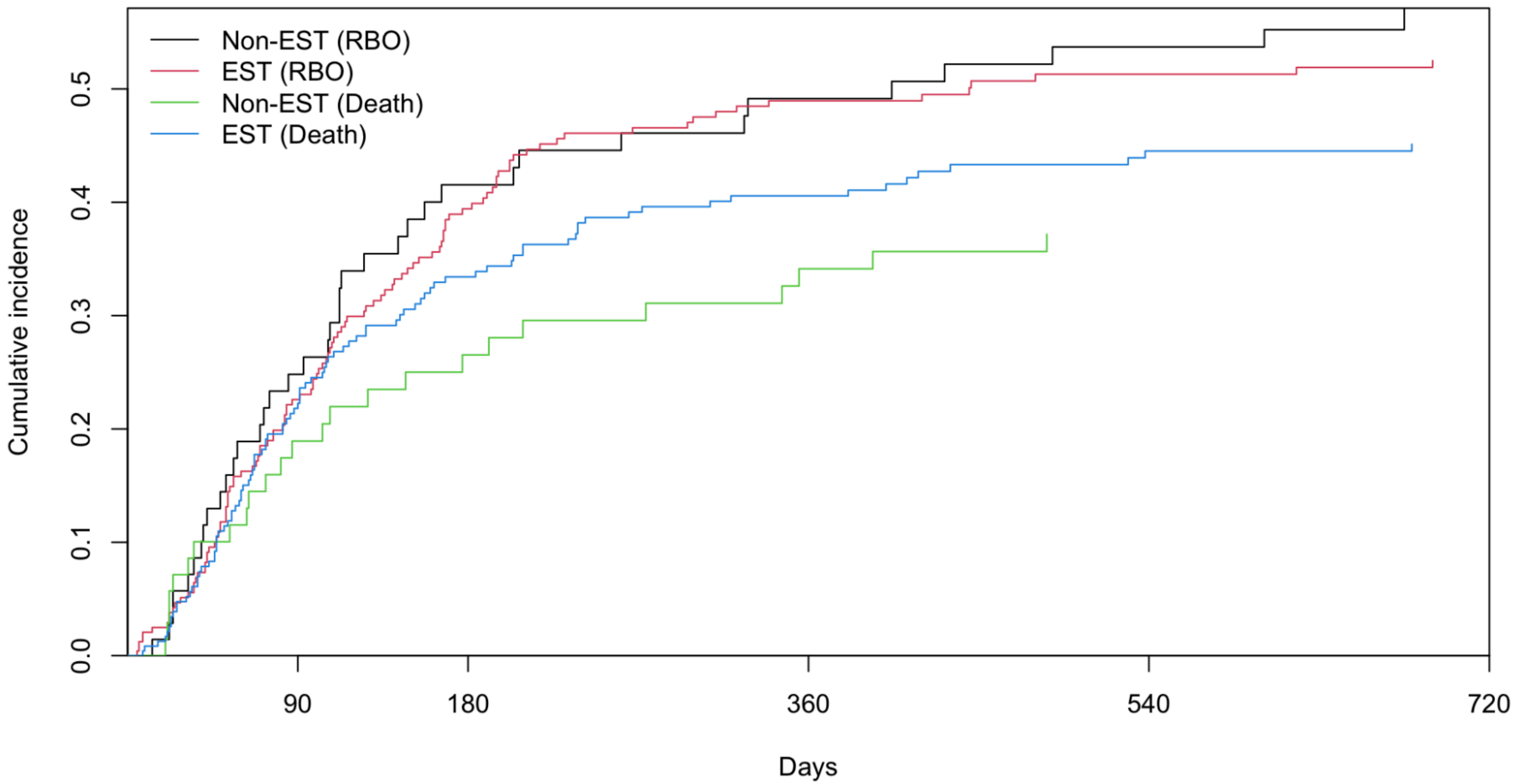

Figure S2. Kaplan–Meier curves for TRBO in clinically successful cases with SEMS, stratified by EST status. / Subgroup analysis restricted to patients treated with SEMS. Statistical values, including median TRBO and log-rank p-values, are shown within the figure.

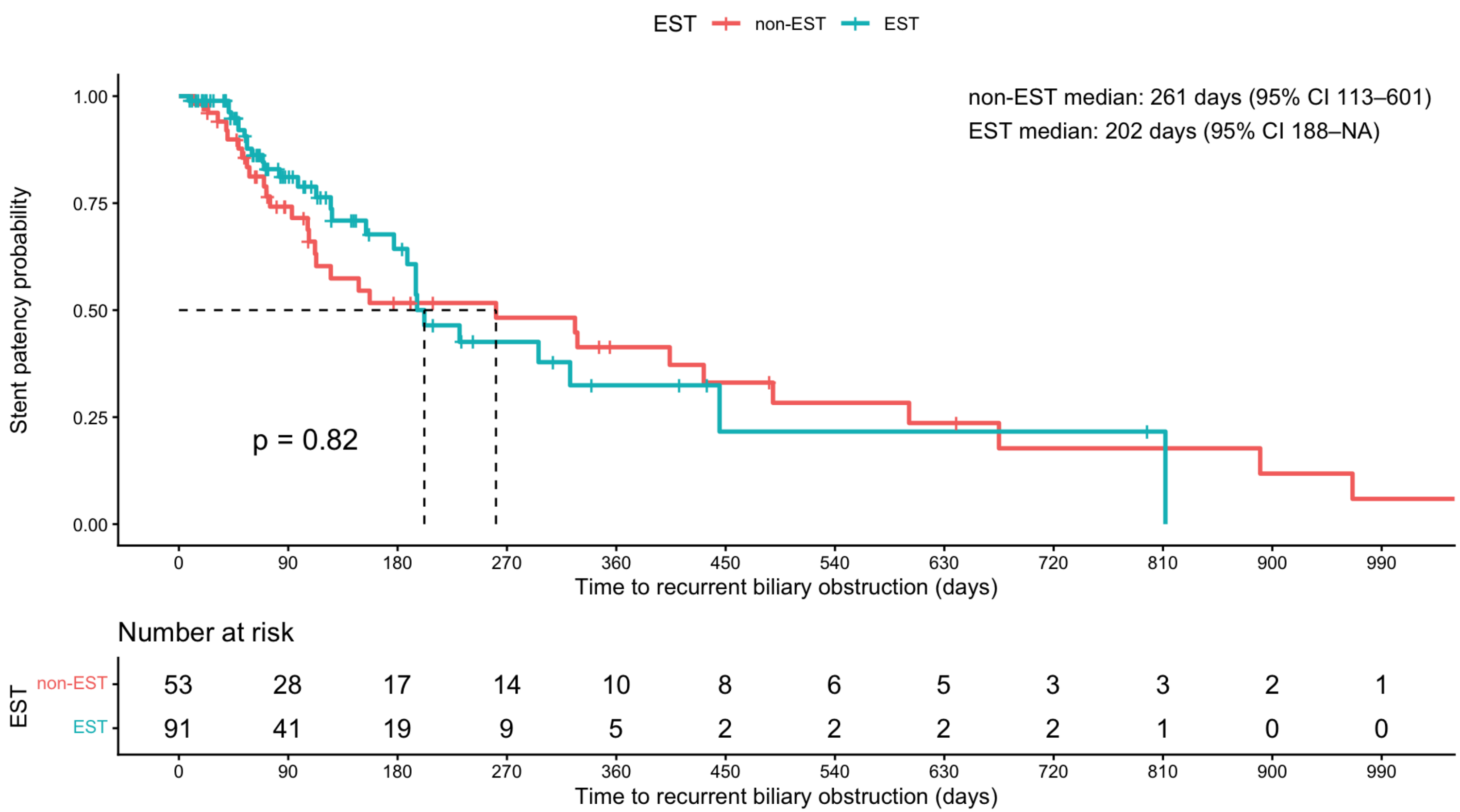

Figure S3. Kaplan–Meier curves for TRBO in clinically successful cases with PS, stratified by EST status. / Subgroup analysis limited to patients treated with PS. Statistical results are displayed within the figure.

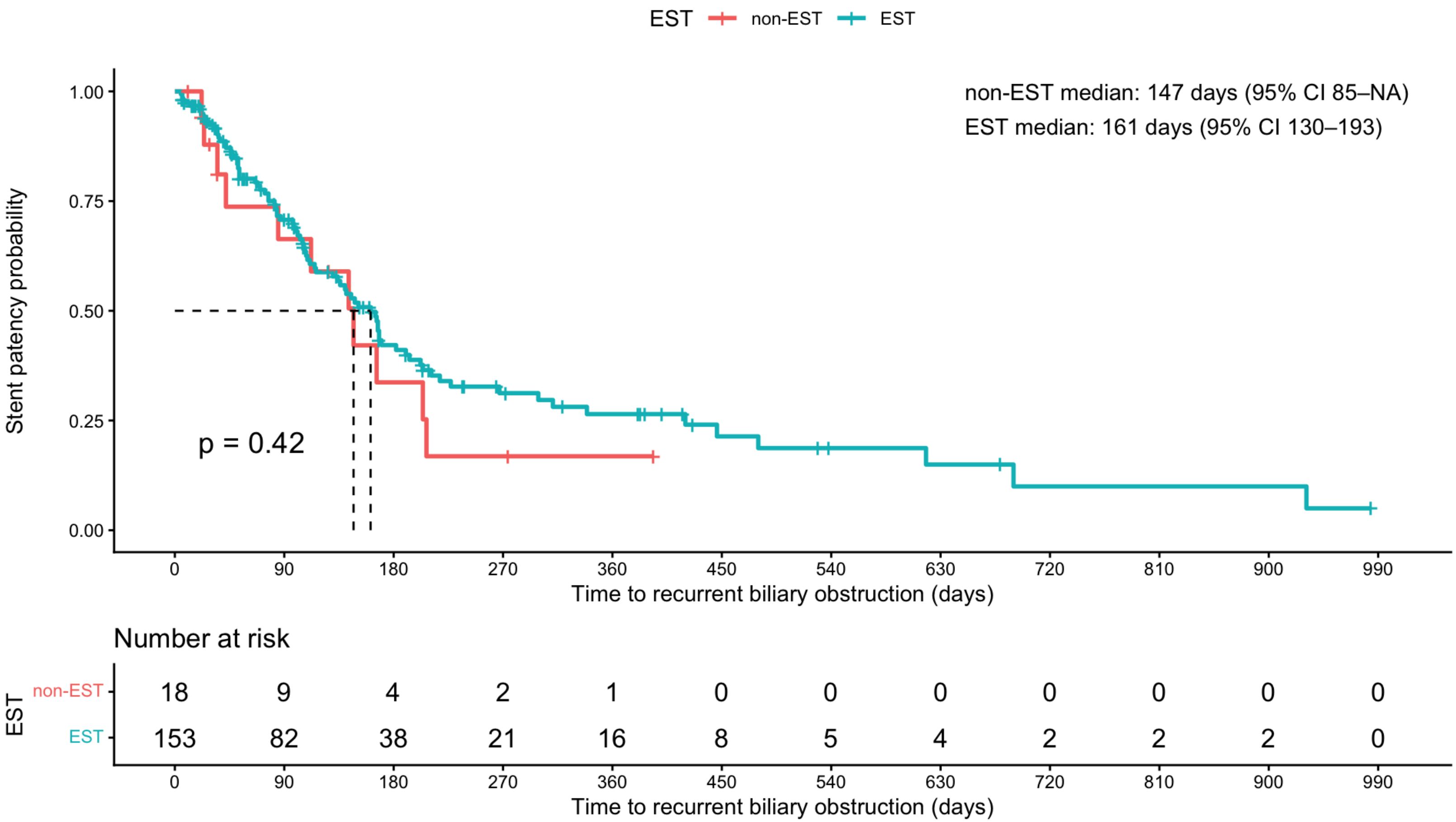

Endosc Int Open | DOI 10.1055/a-2962-1556 | © The Author(s).

Figure S4. Kaplan–Meier curves for TRBO in clinically successful cases with Bismuth type I–II disease, stratified by EST status. / Early-stage hilar strictures (Bismuth I–II). Statistical results are shown within the figure.

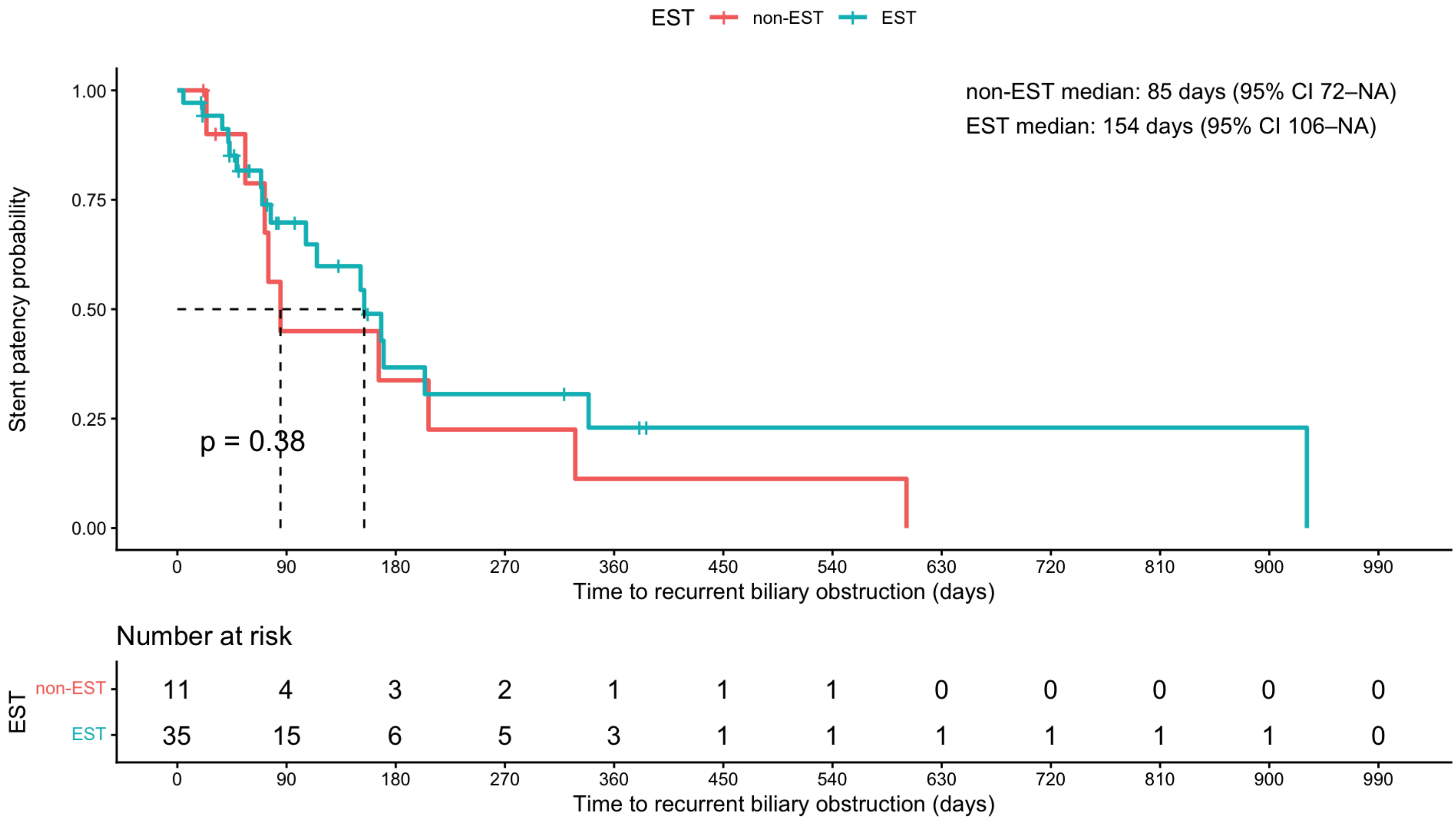

Thieme

Figure S5. Kaplan–Meier curves for TRBO in clinically successful cases with Bismuth type III–IV disease, stratified by EST status. / Advanced hilar strictures (Bismuth III–IV). All statistical values are provided within the figure.

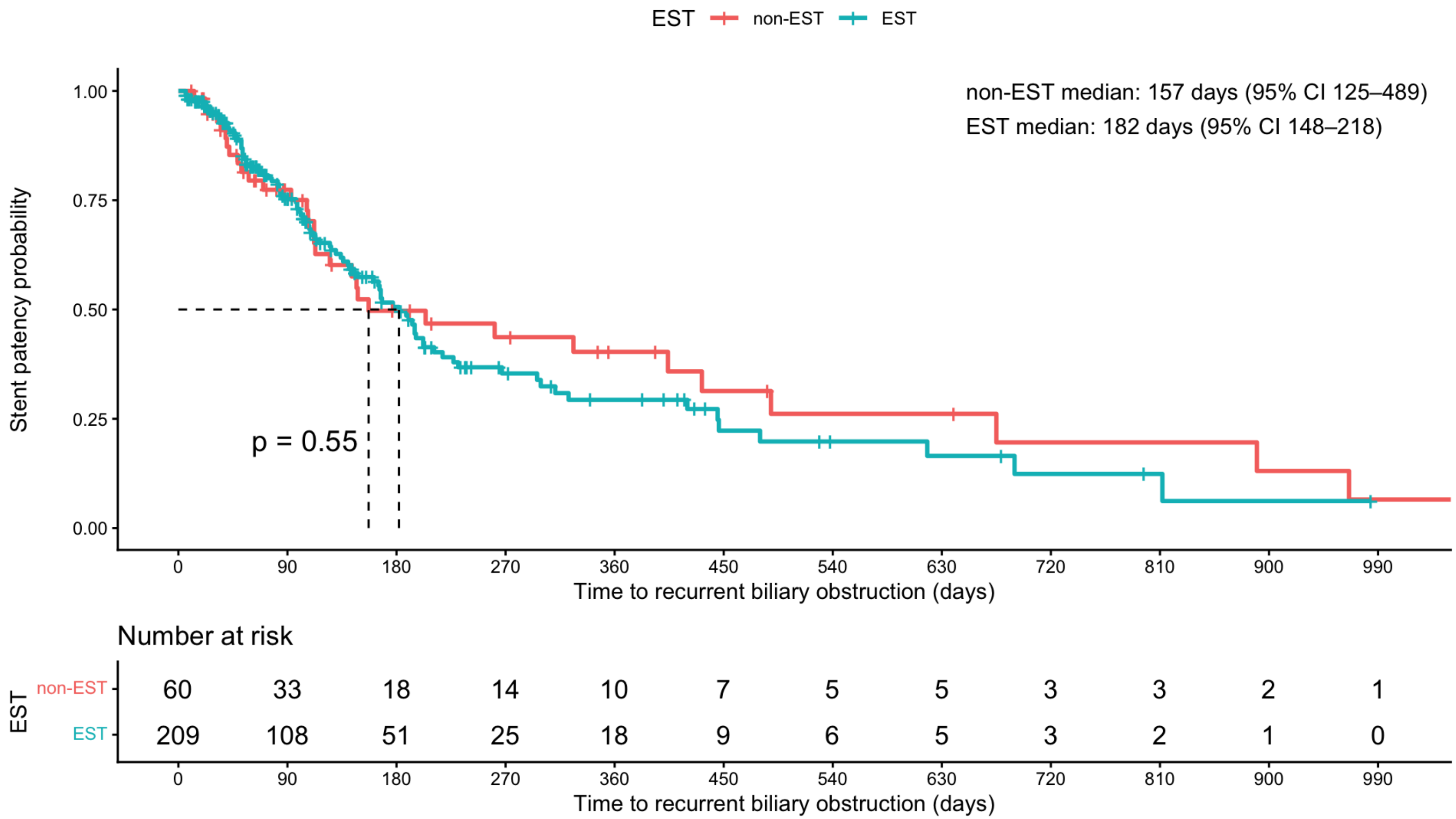

Thieme

Figure S6. Kaplan–Meier curves for TRBO in clinically successful cases with drainage <50% of liver volume. / Subgroup analysis of insufficient drainage (<50%). Statistical values are shown within the figure.

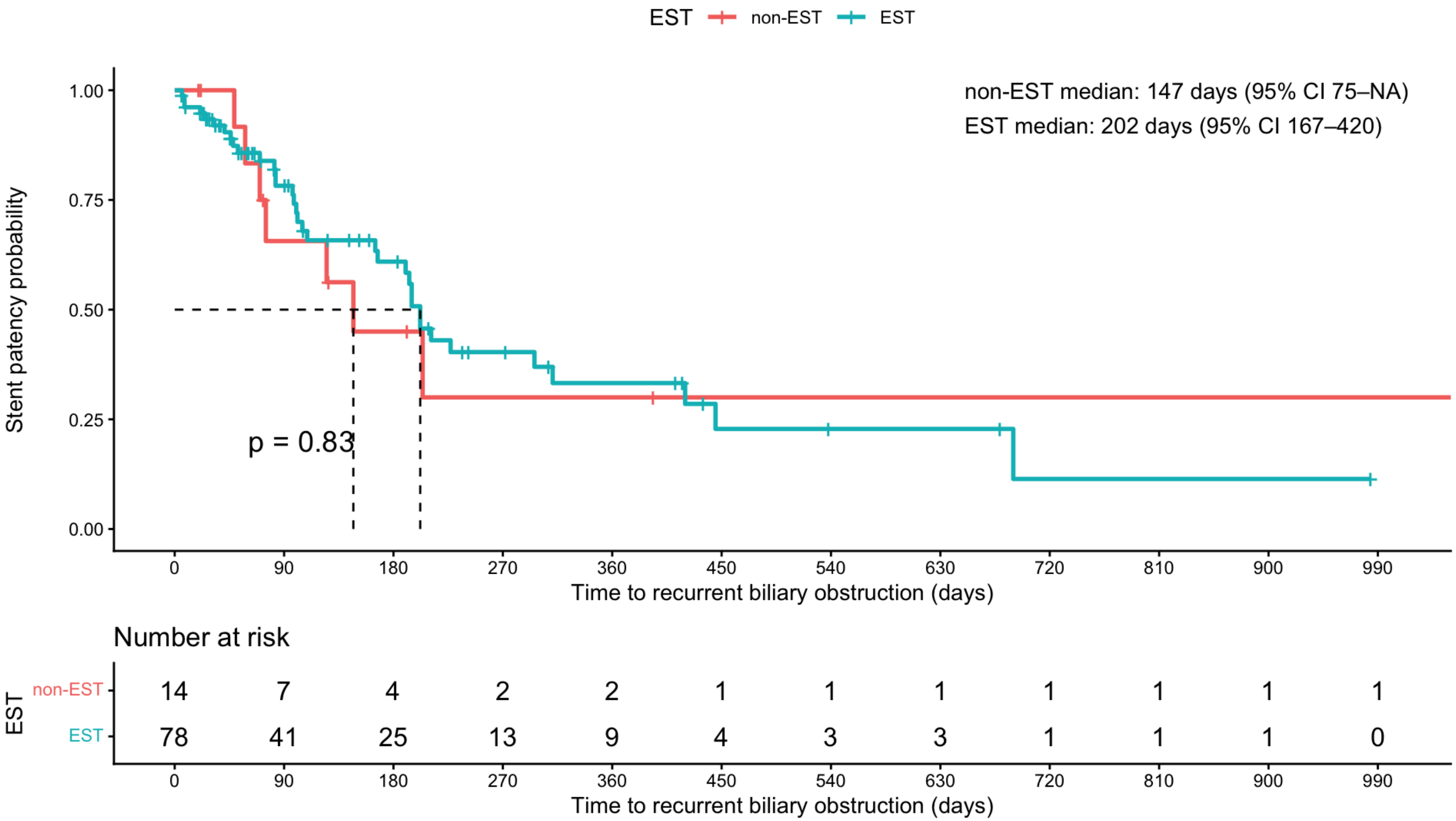

Figure S7. Kaplan–Meier curves for TRBO in clinically successful cases with drainage ≥50% of liver volume. / Subgroup analysis of adequate drainage (≥50%). Statistical results are displayed within the figure.

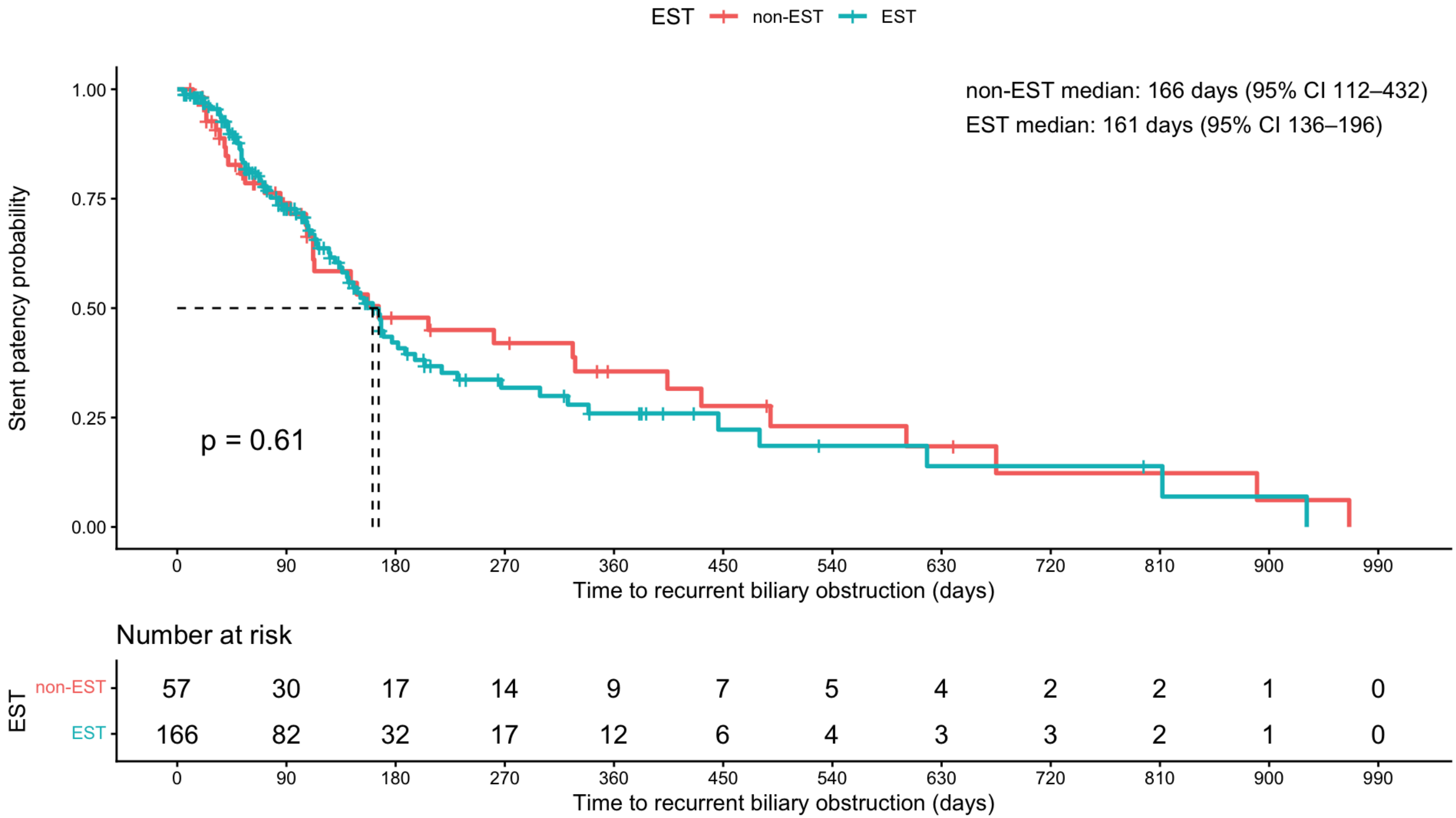

Endosc Int Open | DOI 10.1055/a-2962-1556 | © The Author(s).

Figure S8. Kaplan–Meier curves for TRBO stratified by stent type (PS vs. SEMS). / Comparison of stent patency between plastic and metal stents. Median TRBO values and log-rank p-values are shown within the figure.

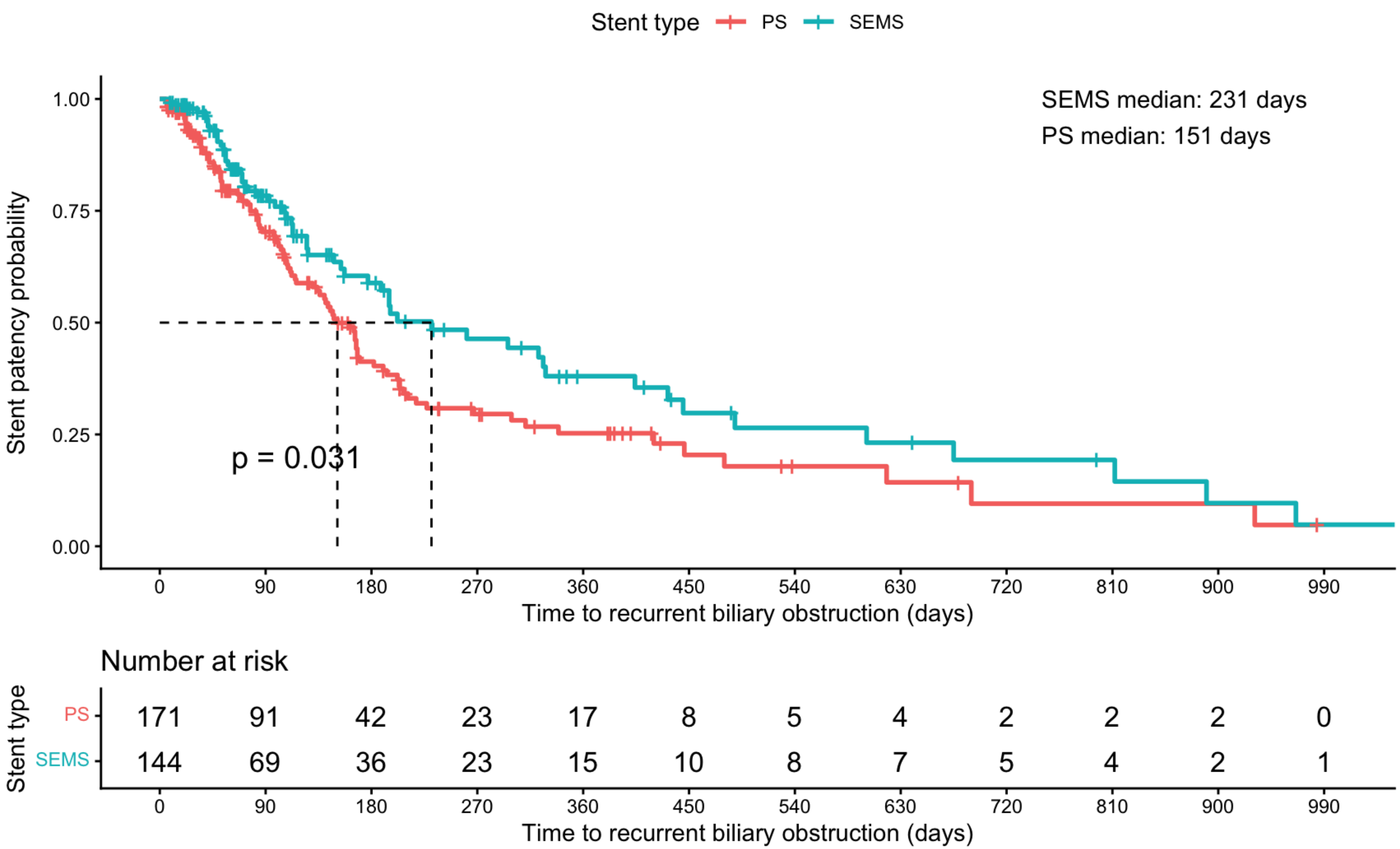

Supplement: Supplementary file 1 [file 10-1055-a-2962-1556_29653934.pdf]
